# (IN) Access to Artificial Limbs: the Patient's Perspective According to the War Amps of Canada

**DOI:** 10.33137/cpoj.v4i2.35972

**Published:** 2021-09-21

**Authors:** A. Petlock, K. DiMario

**Affiliations:** Advocacy Program, The War Amputations of Canada, Ottawa, Canada.

**Keywords:** Health Economics, Prosthetic Care, Artificial Limb, Funding Agencies, Insurance, Public Healthcare

## Abstract

Funding agencies, both public and private, do not adequately meet the needs of Canadian amputees. This often leaves amputees without access to appropriate prosthetic care due to outstanding balances that are to be paid out of pocket, or by charitable organizations. There are several factors that result in these inadequacies. As healthcare is a provincial responsibility in Canada, provinces and territories have the authority to create individual public regimes, each with their own weaknesses. In fact, there are a few provincial regimes which do not include prosthetic funding at all. Private healthcare is meant to offset the remaining balance; however, their lack of knowledge regarding amputation has resulted in the creation of policies with ambiguous language, limiting the funding available for prosthetic care. Attitudinal barriers and missed legislative opportunities further exacerbate the shortcomings of prosthetic funding provided by public and private funding agencies, requiring action.

## INTRODUCTION

The average Canadian would be shocked to know that if they or a family member lose a limb, they could be faced with a personal balance of thousands of dollars for even the most basic artificial limb that will restore only a semblance of their previous function.

Insufficient access to funding for artificial limbs is a key barrier faced by Canadians with amputations. Across the country, both public and private funding agencies create and adhere to policies that do not reflect the reality of living with amputation and that, when applied, prevent amputees from being able to access prosthetic care that is medically prescribed and essential to their everyday functionality.

For those who cannot afford to pay thousands out of pocket, few alternatives exist. For many, crowdfunding has proven necessary, a veritable canary in the coal mine pointing to a distressing state of affairs for Canada's healthcare system.

It is also only a stop-gap solution, given that amputees will understandably be reluctant or simply unable to repeatedly appeal to friends and family for the subsequent replacement limbs they will need during their lifetime.

It is unimaginable in the 21st century that seriously disabled amputees would have to rely on their families and communities in this way to cope with the basic financial requirements.

The purpose of this paper is to highlight the nature of the issues in the public and private realms, describe the attitudinal and legislative barriers that perpetuate these issues and suggest mechanisms for how patients, professionals and the public can work together to improve access to funding for artificial limbs, and, as a result improve the lives of Canadian amputees.

## 1. FUNDING FOR ARTIFICIAL LIMBS IN CANADA: A MULTI-LEVEL BATTLEFIELD

Across the country, there are over 40 public and private agencies that provide funding for artificial limbs. These range from federal, provincial and municipal governments to workers compensation regimes, employment and private insurance companies and charitable agencies, including The War Amps of Canada. Although there are various agencies that provide funding towards the cost of artificial limbs, the funding received from even one source does not adequately support the average amputee in Canada.

Generally, persons with amputations have three potential routes to access funding for prosthetic care (excluding crowdfunding): provincial healthcare funding, private insurance, and charitable sources. Other than small grassroots and locally based amputee groups like the Ottawa Amputee Society, The War Amps is the only large charitable organization in support of amputees in Canada.

The War Amps, a charitable organization that does not receive government grants, fills the gaps in funding where it can; however, as a charitable organization that relies on public donations, these funds can only go so far. As such, The War Amps mandate stipulates that funding can only be accessed after other government and insurance agency contributions are exhausted.

Unfortunately, provincial healthcare and private insurance regimes miss the mark, leaving large outstanding balances to be funded by the amputee themselves or a charitable organization.

### 1.1. Public Healthcare in Canada

Due to Canada's constitutional framework, healthcare is a provincial responsibility. The Canada Health Act mandates that all provinces must cover the cost of “essential healthcare services”, defined as those administrated by doctors in hospitals. Anything outside of essential services are deemed to be provided “at the discretion of the province”.

As a result, provinces and territories in Canada have used their discretionary authority to create no less than 13 different public regimes for covering the cost of prosthetic care (one for every province and territory). Each one of these systems fails, in a unique (and creative) manner to adequately respond to the reality of living with amputation and relying on prosthetic care. As a result, the lack of access to quality mobility aids, devices and assistive technology at an affordable cost remains a barrier to accessibility for Canadian amputees. See **Table 1**, for an overview of how these systems fail to meet the mark.

**Table 1:** Provincial Funding Issues.

Province(s)	Description of funding issues
**Newfoundland and New Brunswick**	No funding available unless in receipt of social assistance. Working residents receive no provincial support for artificial limbs. For those receiving social assistance, the maximum provincial contribution is often insufficient to cover the total cost of the prosthesis.
**Prince Edward Island**	No policy exists. Amputees receive coverage if they receive care at the one public prosthetic clinic in the province. No coverage is available if they receive care elsewhere.
**Nova Scotia**	Limited funding is available. If prosthetic care is received at the one publicly run centre in the province, then full coverage is possible. Wait times are extensive. When care is received at a private clinic, the province covers approximately 1/3 of the cost.
**Quebec**	Some funding available according to a fee schedule. Fee schedule is extremely outdated and includes items that are no longer prescribed and excludes basic prosthetic components. If persons with amputations require components not listed on the fee schedule, then they receive no provincial funding. All components must be from their approved list or no coverage is received.
**Ontario**	Some funding available according to a fee schedule which has not been updated since 2006. Fee schedule includes items no longer prescribed and excludes basic prosthetic components. Still, Ontario claims to cover 75% of prosthetic care costs, but since the fee schedule is badly outdated, it is often typically 10-30%.
**Manitoba**	Funding is available. Lack of transparency for what is covered. No written policy is available. No updates have been made to the fee schedule in many years and there is no mechanism for ongoing updates to policy or fee schedule.
**Saskatchewan**	Funding is available and generally covers what is required for amputees. Care is only offered in two centers in the province leading to long wait times. If care is required sooner, often amputees choose to go out of province (to Alberta). If they do so, coverage can be less than 1/3 of the cost.
**Alberta**	Funding is currently available. On Dec 23, it was announced that private insurance must be exhausted before provincial support can be accessed, which is unusual, unprecedented and will generate significant complexity in 2021. In addition, fee schedules for reimbursement amounts for prosthetic componentry are outdated and not reflective of current cost structures. Movement toward privatization may bring further regression and spur some of the issues with the insurance industry we are familiar with.
**Yukon and the Territories**	Persons with amputations living in remote communities' travel to the nearest prosthetic clinic in a neighboring province. Territories generally cover the cost of care and the travel out of province. The issues arise when we attempt to locate policies or standards which govern the decisions to approve or deny prosthetic care: none appear to exist. Hence, amputees are faced with ambiguity about whether the territory will cover the cost of care. We have seen circumstances where standard care seems to be arbitrarily denied, with no policy basis.
**British Columbia**	Funding is limited to items that restore “basic functionality”. This term is ambiguously defined, narrowly applied, and fails to realize that artificial limbs do not provide functionality equivalent to the missing limb. As a result, prosthetic care and componentry that is considered “standard” by the prosthetic profession, industry and medical community are arbitrarily excluded. For example, myoelectric hands, which have been an integral aspect of prosthetic care for over 30 years are not covered. Due to the ambiguous definition, there is uncertainty regarding what will be and what will not be covered from fitting to fitting.

### 1.2. Case examples

#### Ontario

The outdated fee-schedule utilized by Ontario's Assistive Devices Program (ADP) leaves many amputees with a large outstanding balance to fund out-of-pocket. See **Table 2** and **Table 3** below for an overview of the lack of coverage provided by ADP.

**Table 2:** ADP Coverage for an above knee prosthesis with a microprocessor knee unit.

Cost of Prosthesis:	$56,102.42
ADP Amount Covered:	$12,286.00
Remaining Balance:	$43,816.42
**Percentage of ADP Coverage:**	**21.9%**

**Table 3:** ADP Coverage for the replacement of a left below elbow myoelectric socket due to growth.

Cost of Prosthesis:	$7,701.06
ADP Amount Covered:	$2,188.00
Remaining Balance:	$5,513.06
**Percentage of ADP Coverage:**	**28.4%**

#### Saskatchewan

A five-year-old bilateral above the knee child amputee was unable to receive timely and effective treatment in Saskatchewan. They were forced to seek care at a centre with more experience with children and multiple amputations out of province. As prosthetic treatment was obtained out-of-province, the province offered reimbursement just over $6,000 towards the total cost of the prescribed bilateral transfemoral prosthesis just over $20,000.

#### British Columbia

The British Columbia Ministry of Health issued approvals for a number of amputees to receive osseointegration surgery in Australia. After they received the surgery, they returned to BC to find that the province refused to cover the cost of their prosthesis. Pharmacare, a branch of the Ministry of Health explained that there was no policy to cover the cost of osseointegration prosthetic care. The amputees who received the surgery, paid for by the province, were left with no coverage for their prosthesis for nearly a year.

Most provinces offer some coverage for artificial limbs, but each system has serious flaws, which move away from a patient centered approach, and regularly negatively impact the health and well-being of a very vulnerable group of Canadians, persons with amputations. Amputation and prosthetic care are a complex and highly specialized area of healthcare. Thus, provinces may be generally unaware of how badly they are failing Canadian amputees. For this reason, it is important for public funding agents to be informed of the reality of living with amputation, and the ways in which their failures impact amputees.

### 1.3. Private Funding for Prosthetic care

To supplement inadequate provincial healthcare funding, the insurance industry plays a large role in providing funding for artificial limbs in Canada. Unfortunately, the insurance industry lacks federal or provincial legislation which builds in protections for vulnerable groups like amputees who require insurance funding for adequate prosthetic care. Hence, the diversity of insurance policy framework is even more vast than public funding frameworks. As the variety of insurance policy language that pertains to artificial limb coverage is studied, findings suggest that the arrow misses the target by immeasurable margins, with insidious results. **Table 4** highlights the arbitrary language utilized in insurance policies which contribute to inadequate prosthetic funding.

**Table 4:** Arbitrary Policy Phrasing.

Policy Phrasing	Explanation
“One limb for life”	Children grow, people have weight fluctuations and components break or wear out. People need replacement artificial limbs roughly every three years. One artificial limb for life is not reflective of the reality of amputation and prosthetic care.
$1,000 maximum	With costs for prostheses ranging from $8,000 to $100,000, this amount is grossly insufficient.
No direct billing or “assignment of benefits”	Few people have $8,000 to $100,000 in liquid assets or credit that they can use to purchase their prosthesis and then wait for reimbursement. For this small demographic, there must be some arrangement where the provider can be paid directly.
“Myoelectrical limbs are excluded”	Myoelectric hand technology is over 40 years old, yet this clause remains common. We suspect that these exclusions were put in place when this technology was new, but after 40 years, it is time for an update. In application, insurers will commonly deny other prosthetic care on the ground that it is myoelectric, when it is not (such as a microprocessor-controlled knee unit). A myoelectric-controlled prosthesis is an externally powered (i.e. powered by battery) artificial limb that uses the existing muscles in a person's residual limb to control its functions; however, a microprocessor-controlled knee unit is a body-powered component (i.e. the forces and movement generated come from the user of the device) that allows the knee position to change slightly by adjusting hydraulic resistance levels to support stability when standing, and when on slopes and/or uneven terrain.
Unreasonable and rigid replacement frequency limits: 5 years, no exception	A lot can happen in five years and these policies offer no room for accommodation. If an amputee experiences volume fluctuation due to a pregnancy, a revision surgery or growth (as in the case of children), they may be precluded from receiving their insurance support for a number of years.
“Subject to usual and customary limits”	This is perhaps the most pernicious phrase to the amputee seeking private insurance to help cover the cost of their prosthetic care. Often, insurers will approve coverage for prosthetic care “subject to usual and customary limits” as defined by their internal polices. In application, this means that an amputee seeking coverage for their $60,000 prosthesis, which may have coverage of 80% written in their policy, may be shocked when they submit their receipt to insurers and the insurer indicates that $5,000 is the “usual and customary limit”. The insurer will not pay a penny more.

## 2. ATTITUDINAL BARRIERS

In studying this issue, findings suggest a number of key attitudinal barriers that act to compound, perpetuate, and reinforce insufficient access to funding for artificial limbs.

Both the Canadian public and key stakeholders including insurers, and government agents exhibit a lack of understanding of a very complex area of health care and medicine, underestimate the cost of prosthetic technology, and assume that prosthetic technology is more advanced than it is, also known as Sci Fi Syndrome.

### 2.1. Lack of Understanding into a Complex Area of Healthcare

Government agencies and insurance companies do not fully comprehend the impact of amputation and the role the prosthesis plays in reducing the incidence of other medical conditions that can develop with amputation. Adequate prosthetic care will also assist in restoring some of the functionality required for them to access services in, and contribute to, their community and workplace. If an amputee does not have access to the proper prosthesis or develops repetitive strain injuries as a result of a lack of appropriate prosthetic care, the potential cost to the governments and insurers could be immense.^1,2^

While the up-front cost of an appropriate, medically necessary prosthesis appears expensive, in the long term it will save costs.^3^ Prostheses have been demonstrated to increase safety and security, and to reduce the incidence of the comorbidities associated with amputation. Subsequently, a decrease in comorbidities translates to a decrease in the costs associated with those comorbidities, which include but are not limited to expensive medication for mental health and pain management, paramedical treatments, treatment for injuries caused by falls, the cost of home modifications, vehicle modifications and daily living aids, as well as additional income replacement costs as the individual is not able to return to work without the appropriate prosthesis.

Public and private funding agencies do not realize the above when they build limiting prosthetic policies or issue coverage denials. Indeed, the choice is to pay now for the prosthesis, or pay later for the comorbidities. Of course, it is the amputee who pays the most.

### 2.2. Underestimate the cost of Prosthetic care

The Canadian public is generally unaware of the often prohibitively high cost of artificial limbs. They assume that costs are only a fraction of the actual cost and do not understand the unique nature of the prosthetic industry. For example, prostheses have a very custom nature to reflect the needs of each individual amputee and their level of amputation, and the engineering of prosthetic components requires significant research and development to ensure functionality.

As a result of this lack of understanding, public and private agencies balk at the cost. “Sticker shock” can trigger denials, and this coupled with Sci Fi syndrome, described below, creates attitudinal barriers to access funding for care.

In addition, the Canadian public often assumes that artificial limbs are fully covered by provincial funding agencies. As Canadians give credit to provinces for care they do not provide, this “credit” further reduces the incentive to make meaningful improvements to prosthetic funding.

### 2.3. Sci Fi Syndrome

Hollywood and the media have raised the expectation of what is possible in prosthetic technology in the eyes of the public, the amputee and their support system, and the funding agencies. All too often, the realities fall short of these expectations, which can have a devastating impact on the amputee and their rehabilitation. The images, the terminology, the stories, and the hype all contribute to the unrealistic expectations.

A number of movies and television shows have featured artificial hands that have more basis in special effects than real prosthetic technology available to the consumer: Star Wars; Robocop; Terminator; The Six-Million Dollar Man, and more. Through entertainment in television, these portrayals set false expectations of the functionality of prosthetic devices, allowing us to ignore the limitations of them. As a result, given the lack of familiarity with prosthetic limbs or with amputation, the public and policy decision-makers often assume that prosthetic limbs provide more functionality than the reality. Sometimes, even assuming that they can offer functionality “greater than the real limb”.

In response to this assumption, decision-makers often assert that the amputee only needs “a basic limb” and not anything “sophisticated”. They do not realize that in truth, no technology available today comes close to replicating functionality lost with the loss of a limb.

The “Sci Fi syndrome” mentality negatively affects amputees by limiting access to technologies which will prevent them from falling, reduce overuse and strain injuries, or help them to maximize their functional ability.

In order for amputees to have access to funding, we must overcome these attitudinal barriers held by the Canadian public, and public and private funding agencies, which are reinforced by Hollywood and the media portrayals.

## 3. LEGISLATIVE OPPORTUNITIES

Current legislative frameworks that could ensure access to funding for prosthetic care are underutilized. Underutilized legislative frameworks is a contributing factor to the failure of the Canadian system to provide appropriate prosthetic funding. Despite Canada's role as a signatory to the United Nations Convention on the Rights of Persons with Disabilities, and recent steps to enhance and define accessibility legislation, Canada still lags significantly behind other comparable countries and persons with amputations continue to be unprotected by high level legal mechanisms.

### 3.1. United Nations Convention on the Rights of Persons with Disabilities

Internationally, it is The War Amps' position that Canada is in violation of its international obligations under the United Nations Convention on the Rights of Persons with Disabilities. Article 20 of this convention has set out that state parties must take steps to facilitate access to quality mobility aids, devices, and assistive technologies, including making them available at affordable costs.

“*States Parties shall take effective measures to ensure personal mobility with the greatest possible independence for persons with disabilities, including by:*

- *Facilitating the personal mobility of persons with disabilities in the manner and at the time of their choice, and at affordable cost;*
- *Facilitating access by persons with disabilities to quality mobility aids, devices, assistive technologies and forms of live assistance and intermediaries, including by making them available at affordable cost;”* ^4^

Further, article 32 states that State Parties will:

- *“[Provide], as appropriate, technical and economic assistance, including by facilitating access to and sharing of accessible and assistive technologies, and through the transfer of technologies.”* ^5^

Canada ratified this Convention in 2010, while seeming to completely overlook this obligation. Amputees, and others who rely on assistive technology for their mobility, do not have access to the mobility aids they need at an affordable price. Canada's failure to appropriately fund artificial limbs for amputees is even more shameful when we consider that the World Health Organization (WHO) has identified artificial limbs as a “Priority Assistive Product” through the GATE Initiative (Global Co-operation on Assistive Technology). The Priority Assistive Product list serves as a model for member states to build their own priority areas and implement by priority. Also included on this list are hearing aids, wheelchairs, communication aids, spectacles, pill organizers and memory aids, among others.

In collaboration with the Convention, the WHO is clear that assistive technologies like artificial limbs should form an integral part of universal health coverage for State Parties who have ratified the Convention. As we know, this is simply not so in Canada. If Canada were to truly follow through on their commitment as a signatory to this convention, it is our position that they would be obligated to make sweeping changes to funding for artificial limbs across the country. As it stands, this Convention remains an important and underutilized tool to improve funding for artificial limbs in Canada.

### 3.2. Accession to the Optional Protocol to United Nations Convention on the Rights of Persons with Disabilities

As of December 2018, Canadians can make a complaint directly to the United Nations if it is felt that their rights as indicated by this convention have been violated.^6^ If the United Nations Committee feels the complaint has merit, they can conduct an investigation and make an order to compel the state party (the country) to comply.^7^

Before this tool for change can be leveraged, we must “exhaust all domestic options”, which means we must pursue opportunities for change and improvement at the provincial and federal level. If this is done with no improvement, then a complaint can be mounted to the United Nations.

Hence, as part of The War Amps' Crusade for Reform for prosthetic funding, we are holding all funding agencies to account and working to advise of the issue and, case by case, challenge inadequate funding. In the next few years, if we do not see meaningful change, we intend to appear before the United Nations Committee on behalf of Canadian amputees.

As a registered Non-Governmental Organization within the Economic and Social Council of the United Nations, The War Amps has a long history of leveraging United Nations Convention Optional Protocols to ensure that the rights of Canadians are upheld. Since the First World War, we have fought to protect the rights of amputees and veterans and address the inequities they face. In that time, we have taken on many important battles in support of amputees and our veterans, including: Hong Kong Veterans and Victims of Thalidomide.

#### Hong Kong Veterans

In 1987, The War Amps, in association with the Hong Kong Veterans' Association of Canada, petitioned The United Nations Commission on Human Rights to demand compensation to Canada's Hong Kong Prisoners of War following the “gross violation of human rights” committed by the government of Japan during the Second World War, which has caused devastating and lifelong health impacts.

The government of Japan did not respond favourably. Thus, The War Amps targeted the Canadian government for its failure to protect the interests of the Hong Kong veterans as part of the Peace Treaty entered into between Allied countries (including Canada) and Japan following the Second World War. Hence, The War Amps initiated a Communication under the Optional Protocol of the International Covenant on Civil and Political Rights alleging a form of discrimination exercised by Canada in its failure to protect the interest of the Hong Kong veterans' prisoners of war against Japan.

The War Amps successfully entered into negotiations with the Canadian government and worked out a financial settlement which resulted in appropriate compensation being paid in the form of an ex-gratia payment from the Canadian government to the individual Hong Kong veterans.

#### Victims of Thalidomide

The War Amps, along with the Thalidomide Victims Association of Canada, also sought proper compensation from the Commission on Human Rights for Canada's Thalidomide victims (survivors) as a direct consequence of the Federal government's distribution of the drug in Canada. Thalidomide was administered to pregnant women in Canada, which resulted in well over 100 children being born with birth defects and serious medical issues, which they continue to confront today.

In September 1989, The War Amps petitioned the United Nations Human Rights Committee under the Optional Protocol pursuant to the International Covenant on Civil and Political Rights. The petition led to direct negotiations with the Federal government and, more particularly, the Ministry of Health, resulting in a settlement which addressed the plight of the Thalidomide survivors to that point in time.

Unquestionably the triggering of the Optional Protocol under the International Covenant on Civil and Political Rights was a key element to having the Thalidomide victims claim recognized by the Canadian government.

In both instances, The War Amps exhausted legal remedies in Canada, and yet, no justice had been served. It was only by leveraging these Optional Protocols that these very vulnerable groups received the support and compensation they needed to move forward after such devastation.

### 3.3 Federal Government Inaction

There are many ways the federal government, in conjunction with the provinces, can ensure that Canada is meeting its international obligation to provide affordable access to prosthetic care. They must:

1. Set a national standard for appropriate prosthetic funding at the provincial level.
2. Build legislation to prevent insurers from being able to create and sell insurance policies that have arbitrary caps on prosthetic funding.
3. Include considerations for access to funding into Accessibility legislation.

#### A national standard for prosthetic funding

As evidenced from the above, Canada is lacking a national standard which facilitates access to assistive technologies at an affordable cost, for persons with disabilities. Such standards have been implemented in other countries. This includes but is not limited to Germany, Australia, and England. Germany transitioned to a national standard for prosthetic funding in 2004,^8^ and Australia initiated the process of implementing a national standard in 2019.^9^ As for England, an executive body was created in 2012, NHS England.^10^ Although NHS England provides a national standard for prosthetic funding,^11^ such a standard may have been prior present to its establishment in 2012.

Though a provincial power, the Canada Health Act sets the national standard for physician services provided in hospitals. Assistive devices are seldom provided in hospital by a physician. Prostheses are prescribed by a physician, but dispensed by Prosthetists inside a hospital or outside, in a privately run certified clinic. Hence, the provinces may execute discretion on the level of support they decide to provide. Sadly, as we summarized above, provincial funding contributions fall short of the actual cost across the country, with some provinces containing no funding at all.

While constitutionally a provincial power, it is not unfamiliar for the federal government to set national standards in areas of provincial jurisdiction, especially as it relates to health care. In fact, it has largely been regarded as a critical role of the federal government in Canada to set these standards to ensure that Canadian values are upheld. This obligation is set out in the Constitution and is affirmed each time the federal government becomes a signatory to a United Nations Declaration. The responsibility and the obligation are clear: the federal government must ensure that national standards are set and upheld, especially in areas affecting vulnerable people such as those with disabilities, including persons with amputations. It is an accessibility issue and an issue of national importance.

We feel that the federal government has the responsibility and the obligation to be proactive in setting national standards, especially if these national standards affect vulnerable minority groups and groups protected under the Canadian Human Rights Act and are connected to commitments made on the international stage.

#### Build legislation to prevent insurers from being able to create and sell insurance policies that have arbitrary caps on prosthetic funding

Due to the serious lack of adequate funding for assistive technology at the provincial healthcare level, many persons with disabilities, especially amputees, rely heavily on their extended benefits or private insurance to help to ensure that the assistive devices they require are affordable. Sadly, too many of these insurance and extended benefits packages contain arbitrary limits on contributions for essential medical devices, including artificial limbs.

The insurance industry in Canada is, in this way, underregulated. We need legislation, similar to the Statutory Accident Benefits Schedule for motor vehicle insurance, which sets out base limits on what insurance companies must cover for artificial limbs.

The War Amps successfully persuades insurers at the grassroots level, case by case, and through higher level negotiations and educational strategy to demonstrate that it is in their best interest to appropriately cover the cost of prosthetic care. Sadly, without the strong arm of legislation, we do not feel that sufficient and widespread change will occur.

#### Accessibility Legislation

Across the country, at the federal and provincial level, sweeping progress has been made to enact comprehensive accessibility legislation. The definition of accessibility has expanded in recent years from a narrow view focusing on ramps and elevators to digital access and universal design.

In order for accessibility legislation in Canada to truly meet its objectives, the legislation must guarantee the availability of appropriate coverage for artificial limbs for all Canadians who require it.

Assistive technology, including prosthetic care, is a critical element of accessibility for amputees. Without these tools, persons with disabilities are barred from completing their activities of daily living, as well as accessing communities and workplaces. The disability community needs a standard which facilitates affordable access to assistive devices, as without this, accessibility will not be achieved. We believe that, through accessibility legislation, both federal and provincial governments have the major responsibility to set and uphold an appropriate standard for artificial limbs, as per their commitment to accessibility, the United Nations and all Canadians.

## DISCUSSION

Federal and provincial funding agencies, as well as private insurers provide insufficient funding for prosthetic care. Their prosthetic funding policies are missing the mark and failing to address the reality of living with amputation. Each province and territory have their own regime, failing in their own unique way to adequately respond to the reality of living with amputation and relying on prosthetic care. In turn, we see vast diversity of insurance policy frameworks pertaining to artificial limbs which perniciously miss the mark, leaving the amputee without the insurance coverage they thought they had paid for. After the traumatic loss of a limb, if an amputee cannot afford to purchase their prosthesis, they are re-victimized, as navigating the repeated red tape, denials and confusion of the process can re-trigger the trauma and direct focus to the loss.

Attitudinal barriers and missed legislative opportunities compound the issue. Both the Canadian public and key stakeholders including insurers, and government agents exhibit a lack of understanding of a very complex area of health care and medicine, chronically underestimate the cost of prosthetic technology and exhibit Sci Fi Syndrome, which all lead decision-makers to build policies that are not reflective of the reality of living with amputation.

Current legislative frameworks that could ensure access to funding for prosthetic care are underutilized, including the United Nations Convention on the Rights of Persons with Disabilities, which sets out that state parties must take steps to facilitate access to affordable prosthetic care. The enforcement mechanism of this Convention can be leveraged after all Canadian remedies have been exhausted.

The federal government has also failed to set a national standard for appropriate prosthetic funding at the provincial level, enact legislation to prevent insurers from being able to create and sell insurance policies that have arbitrary caps on prosthetic funding, and include considerations for access to funding for assistive devices into accessibility legislation. As such, they are allowing Canada to lag significantly behind other comparable countries. They are allowing vulnerable persons with amputations to be unprotected by high level legal mechanisms.

Hence, holding funding agencies accountable and advocating on behalf of amputees is important in this regard.

## CONCLUSION

Recent years have demonstrated that prosthetic funding provided by public and private funding agents fails to meet the needs of amputees, creating a large barrier to access to care. Both The War Amps and medical professionals are committed to collaborating to respond to the urgent, complex and multi-faceted issue of insufficient prosthetic funding in Canada. Medical professionals can assist further by enrolling all amputees with The War Amps Child or Adult Amputee Programs, challenging issues with insufficient prosthetic funding, and continuing to initiate discussion to educate other medical professionals, the public and the patient population about the issue to generate and leverage the “shock” in response to the serious inadequacies with prosthetic funding in this country.

The issues with access to prosthetic funding are significant, complex, and not well known or understood. As the voice representing the needs of all amputees in Canada, The War Amps is committed to crusading for reform on this issue. With a collaborative and multi-faceted approach, we can continue to move the ball forward to remedy this significant gap. Persons with amputation have experienced significant trauma. A small and vulnerable, yet often resilient demographic, persons who have lost limbs deserve access to prosthetic care. Access to appropriate prosthetic coverage will help restore some of what they lost with the loss of their limbs, without the fear, anxiety and veritable humiliation that accompanies insufficient funding for prosthetic care.

## ADVOCACY FOR AMPUTEES: A CALL TO ACTION

Due to the limitations of the funding regimes available to Canadian amputees, action is required to improve funding regimes for artificial limbs, and subsequently the lives of all amputees. Since 2013, The War Amps has been proactively extending support and sharing expertise with the government and insurers, as well as employers, and works with the profession to improve the standards of funding for artificial limbs through a Crusade for Reform. By educating public health care and private insurance agencies on the necessity of artificial limbs, the goal is to reform and improve the system so that amputees will be able to receive the limbs they need for their independence, safety, and security.

Collaboration between prosthetists, physiatrists, amputees, and charitable organizations will help with the identification of systemic issues in relation to inadequate prosthetic funding in an effort to improve funding for artificial limbs in Canada. Key steps that patients and professionals can take to contribute include:

### 1) Enrolling all amputees with The War Amps Child or Adult Amputee Programs

One barrier to improving prosthetic funding is the lack of data and statistics regarding the number of amputees in Canada. Statistics Canada and other agencies do not collect this information. Hence, by enrolling all amputees, we can help address this knowledge gap by ensuring that data we collect on amputees is as representative as possible as a basis for argument and decision-making in support of prosthetic funding.

### 2) Challenge Inadequacies

If medical professionals, in practice, encounter an issue with funding, whether government or insurance, we encourage them to take steps to challenge it. Across the country, prosthetists have built this step into the support they offer to their patients with demonstrated success, but we understand the administrative burden this can present to a small business.

Hence, The War Amps is available to assist with challenging these issues. We can appeal individual denials, or partial approvals. We can educate insurers, and provincial funding agencies on their inadequacies. We have successfully persuaded a number of funding agencies to apply appropriate funding on appeal through the use of alternative dispute resolution mechanisms (i.e., the submission of letters and appeals to the insurer), and we simply will not relent until we receive adequate response.

In this way, we will continue to exert the required pressure toward change, and when the time is right, use these efforts as evidence towards the need for us to take the next step: to bring this issue into the international arena by way of the United Nations Convention of the Rights of Persons with Disabilities.

### 3) Educate and Initiate

Medical professionals can continue to keep The War Amps abreast of issues they are facing and call on The War Amps for support to help strengthen the overall position and argument in favour of increased prosthetic funding.

Conversations must be initiated between contacts, acquaintances and other medical professionals who work with amputees to educate them on the issue. Most Canadians are unaware of the serious issues with prosthetic funding and, hence assume that appropriate funding is granted to all Canadians. Generating and leveraging this “shock” will help elevate The War Amps' Crusade in the minds of the Canadian public and thus, government agencies.

The three actions listed above are first steps in this major Crusade and may seem minor, but they play keystone roles in improving funding in Canada.

## DECLARATION OF CONFLICTING INTERESTS

The authors declare no conflict of interest.

## SOURCES OF SUPPORT

None.

## AUTHOR SCIENTIFIC BIOGRAPHY

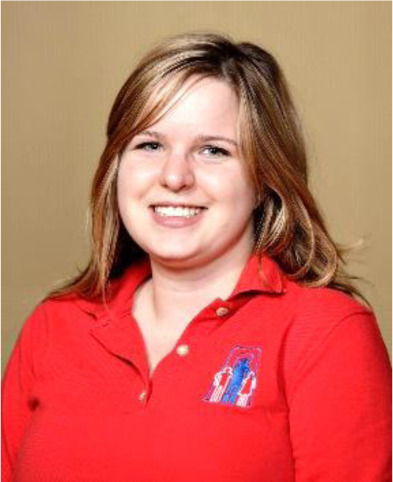



**Annelise Petlock** is a lawyer with an MBA who serves as the Manager of The War Amputations of Canada's Advocacy Program. Annelise has an in-depth understanding of amputation and the whole-body impact it has on the lives of individuals living with amputation, including the role of the artificial limb. The War Amps is a charity devoted to improving the lives of amputees in Canada, including children. The War Amps provides financial assistance for artificial limbs, peer support and information on all aspects of living with amputation.

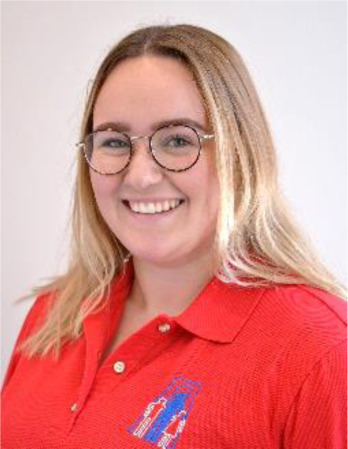



**Keana DiMario** serves as a Case Coordinator of The War Amputations of Canada's Advocacy Program. Keana addresses issues facing amputees across all areas including insufficient prosthetic funding.
